# In search of the optimal MRI marker for progressive supranuclear palsy: a large, single-center, retrospective study on the effect of phenotype, diagnostic certainty and disease duration

**DOI:** 10.1007/s00415-025-13262-2

**Published:** 2025-07-21

**Authors:** Vasilios C. Constantinides, Nikolaos Giagkou, Maria-Evgenia Brinia, Ioanna Kapsali, Georgios Velonakis, Sokratis G. Papageorgiou, George P. Paraskevas, Elisabeth Kapaki, Leonidas Stefanis

**Affiliations:** 1 First Department of Neurology, National and Kapodistrian University of Athens, School of Medicine, Eginition Hospital, 72-74 Vas. Sofias Ave., 11528 Athens, Greece; 2 Research Unit of Radiology, Second Department of Radiology, National and Kapodistrian University of Athens, School of Medicine, Attikon Hospital, Athens, Greece

**Keywords:** Progressive supranuclear palsy, Parkinsonism, MRI, Magnetic Resonance Parkinsonism Index, Midbrain, Volumetry

## Abstract

**Introduction:**

Multiple MRI markers have been introduced as surrogate markers of progressive supranuclear palsy (PSP). Midbrain surface, midbrain/pons surface ratio (M/P) and the Magnetic Resonance Parkinsonism Index (MRPI) have produced high diagnostic accuracy in differentiating PSP from other parkinsonian disorders. A systematic comparison of the diagnostic accuracy of available MRI markers, and the effect of disease duration, clinical presentation, level of clinical certainty on the performance of these markers is lacking.

**Materials and methods:**

In this single-center, retrospective study, 244 subjects were included (80 PSP, 38 corticobasal degeneration, 45 multiple system atrophy, 36 Parkinson’s disease patients, 45 control subjects). All patients underwent a standardized MRI acquisition protocol and automated MRI data preprocessing through Freesurfer for volumetric data. Midbrain distance, surface and volume, superior cerebellar peduncle (SCP) width and volume, and composite markers including the M/P and M/P 2.0 ratios and the MRPI and MRPI 2.0 were measured. The diagnostic accuracy of these markers was calculated, and the effect of clinical phenotype, level of disease certainty and disease duration were investigated.

**Results:**

Surface-based MRI markers were superior to distance- and volume-based markers. Midbrain surface was the optimal MRI marker for PSP (AUC = 0.937; sensitivity 88.8%; specificity 84.2%). MRI markers were more accurate in diagnosing probable PSP vs. possible/suggestive PSP and PSP-Richardson syndrome vs. PSP variants. MRI markers exhibited comparable diagnostic accuracy in early (≤ 24 months) vs. late (≤ 48 months) disease stages. Midbrain atrophy correlated with PSP disease severity as well as ocular motor, gait and bulbar deficit severity.

**Discussion:**

Planimetric MRI markers are optimal for PSP diagnosis. Level of disease certainty (i.e. probable vs. possible/suggestive) and clinical presentation (PSP-RS vs. PSP variants) affect the diagnostic accuracy of MRI markers. MRI markers are useful even in early (≤ 24 months) stages of PSP.

**Supplementary Information:**

The online version contains supplementary material available at 10.1007/s00415-025-13262-2.

## Introduction

Progressive supranuclear palsy (PSP), a 4R-tauopathy, is a rare neurodegenerative disease which is defined neuropathologically by the aggregation of intranuclear hyperphosphorylated tau protein [1]. PSP traditionally manifests with Richardson’s syndrome (PSP-RS), characterized by supranuclear gaze palsy and early postural instability [2]. Neuropathologically, PSP-RS exhibits relatively selective midbrain and superior cerebellar peduncles (SPCs) atrophy [3].

Over the course of the last three decades, neuropathological data have established the great phenotypical heterogeneity of PSP. This clinical heterogeneity is highlighted in the most recently established PSP diagnostic criteria, which recognize eight different clinical PSP phenotypes [4]. These include PSP-RS and seven additional phenotypes which are collectively termed PSP variants and include diverse syndromes such as: PSP with predominant parkinsonism (PSP-P), which may resemble Parkinson’s disease; PSP with progressive gait freezing (PSP-PGF), characterized by early predominant gait freezing; PSP with predominant postural instability (PSP-PI), wherein patients have predominant postural reflex abnormalities, without concomitant supranuclear gaze palsy; PSP with predominant ocular motor dysfunction (PSP-OM), where oculomotor abnormalities are the predominant clinical feature, in the absence of postural instability; PSP with predominat frontal presentation (PSP-F), wherein patients have significant frontal/executive deficits; PSP with predominant speech/language disorder (PSP-SL), wherein patients exhibit an effortful, non-fluent agrammatic speech, and PSP with predominant corticobasal syndrome (PSP-CBS).

In light of this significant clinical heterogeneity, accurate diagnosis of PSP in vivo can be difficult, particularly early in the disease course of PSP-RS patients, in oligosymptomatic cases, in PSP variant patients and in instances of atypical and/or mixed clinical presentations. To this end, multiple MRI markers have been introduced to assist in the early and accurate diagnosis of PSP. These markers conceptually rely heavily on the neuropathologically proven selective midbrain and SPC atrophy in PSP-RS.

These MRI markers can be classified into: a) simple markers, which include measurement of a specific region of interest (e.g. midbrain, SCP) and: b) composite markers, which incorporate multiple measurements in a formula (e.g. midbrain surface to pons surface). In addition, MRI morphometric markers can be classified based on the modality of measurement and can thus be classified into: (a) distance-based; (b) surface-based and: (c) volume-based MRI markers. Simple MRI markers have focused on atrophy of midbrain (anterior–posterior distance, surface, volume) and SCP (width, volume). Composite MRI markers have incorporated pontine and middle cerebellar peduncle (MCP) measurements as reference points, in an effort to attenuate the selective midbrain/SCP atrophy in PSP. These include the pons/midbrain ratio (P/M) [5] and P/M 2.0 [6] as well as the Magnetic Resonance Parkinsonism Index (MRPI) [7] and MRPI 2.0 [6].

Early studies focusing on the use of the anterior–posterior midbrain distance as a potential marker for differentiating PSP from PD and MSA patients have yielded conflicting results, with some studies supporting suboptimal diagnostic accuracy, and a single study supporting diagnostic accuracy comparable to M/P surface ratio (but with moderate AUCs) [8–11]. Midbrain surface on the other hand has resulted in high diagnostic accuracy in discriminating PSP patients from MSA and CBD patients, with > 90% sensitivity/specificity [12, 13], with some overlap however between PSP and MSA patients [5]. Midbrain volume has resulted in high diagnostic accuracy when applied to differentiate PSP from PD patients [14], but suboptimal diagnostic accuracy in the differentiation of PSP from CBD [12, 15]. SCP volume as a PSP marker produced high specificity but moderate sensitivity in a cohort including MSA and PD patients [16].

Regarding composite MRI markers, initial studies supported a high diagnostic accuracy of both the M/P surface ratio and the MRPI in the differentiation of PSP from MSA and PD [5, 7]. Several follow-up studies compared the diagnostic accuracy of these two composite markers, with most studies reporting superior sensitivities/specificities for the MRPI over the M/P surface ratio [17–22]. Few studies however have challenged this view, reporting superior predictive accuracy of the M/P surface ratio compared to the MRPI, particularly in the differentiation of PSP from PD [12], PSP-P from PD [14] and PSP from other parkinsonian disorders [23]. Due to the suboptimal diagnostic accuracy of the M/P and MRPI in the differentiation of PSP-P patients from other parkinsonian disorders [24], the M/P 2.0 and MRPI 2.0 composite markers were introduced, resulting in improved diagnostic accuracy for PSP-P [6]. A recent meta-analysis comparing these markers concluded that the midbrain surface was superior to M/P surface ratio and MRPI in the differentiation of PSP-RS from control subjects [25].

Despite the presence of multiple studies focusing on the application of different MRI markers in the differential diagnosis of parkinsonian disorders, there are certain issues regarding these markers which have not been systematically addressed by the literature to date. More specifically, to date no study has systematically compared established MRI markers of PSP of different modalities (i.e., linear vs. planimetric vs. volumetric). In addition, the possible effect of disease duration, clinical presentation and level of diagnostic certainty on the diagnostic performance of these MRI markers has not been studied.

In an effort to address these issues, we performed a retrospective, single-center study in a large cohort of patients with parkinsonism, with MRI data available based on a standardized MRI acquisition protocol, with the following primary endpoints: (a) comparison of the diagnostic accuracy of MRI markers of different modalities in PSP(i.e. simple vs. composite; distance-based vs. surface-based vs. volume-based); (b) comparison of the diagnostic accuracy of MRI markers in PSP-RS vs. PSP variants; (c) comparison of the diagnostic accuracy of MRI markers in PSP patients stratified by certainty of clinical diagnosis (i.e. suggestive vs. possible vs. probable diagnosis); (d) comparison of the diagnostic accuracy of MRI markers in PSP patients stratified by disease duration. Secondary endpoints were: (a) the investigation of possible imaging-clinical correlations of these MRI markers, and (b) investigation of possible correlations among MRI markers of different modalities.

## Methods

### Study participants

The medical files of all patients consecutively referred from 2014 to 2023 to the “Neurodegenerative Disorders and Epilepsy Ward” of Eginition Hospital were retrospectively reviewed. Patient selection was performed by four of the authors (V.C.C., M-E.B., N.G, I.K.), by use of the existing patient database of our Ward. Each eligible patient file was audited by two authors independently, who reached a final clinical diagnosis based on the clinical data of the file, in accordance with most recent established diagnostic criteria. Patients were included only in cases of agreement between the two auditors. The inclusion criteria for the purposes of this study were: (a) patients fulfilling most recent established diagnostic criteria for Parkinsons’s disease [26], progressive supranuclear palsy [4], multiple system atrophy [27], corticobasal degeneration [28] were included. These criteria were applied retrospectively to patients evaluated prior to the publication of the respective diagnostic criteria, based on their medical files. Imaging data were not taken into consideration for patient diagnosis. Patients with missing data were excluded; (b) availability of MRI data, based on a specific MRI protocol performed at Eginitio Hospital (see Sect. 2.3). In addition to patient groups, a total of 45 control subjects were included. The control subjects had no history of major psychiatric or neurologic disease, had normal neurological examination and normal neuropsychological evaluation (based on performance on the MMSE and FAB tests, taking into account their age and educational level).

The following demographic and clinical data were recorded: (1) sex; (2) age in years; (3) disease duration in months; (4) diagnosis (i.e., PSP vs. MSA vs. CBD vs. PD vs. controls); (5) clinical syndrome: PSP with Richardson’s syndrome (PSP-RS); PSP with predominant parkinsonism (PSP-P): PSP with progressive gait freezing (PSP-PGF); PSP with predominant frontal presentation (PDP-F); PSP with predominant speech/language disorder (PSP-SL); PSP with predominant corticobasal syndrome (PSP-CBS); PSP with predominant postural instability (PSP-PI); PSP with predominant ocular motor dysfunction (PSP-OM); (6) level of diagnosis certainty (i.e., clinically established vs. probable vs. possible vs. suggestive); (7) Unified Parkinson’s disease Rating Scale Part III (Motor Examination; UPDRS-III); (8) Mini Mental State Examination (MMSE); (9) Frontal Assessment Battery (FAB); 10) the 5 words immediate and delayed recall test (5 WR); (11) the 15-point Clox 1 (spontaneous) and Clox 2 (copy) clock drawing test, and (12) the Progressive Supranuclear Palsy Rating Scale (PSPRS) total score and History, Mentation, Bulbar, Ocular Motor, Limb Motor and Midline/Gait sub-scores where applicable and available. Despite the retrospective inclusion of patients for the purposes of this study, all clinical and neuropsychological data in our ward are generated prospectively, based on a semi-structured interview and thorough neurological examination, with systematic recording of relevant symptoms and signs, their date of onset and their course.

### Ethical considerations

The Ethical and Scientific Committee of Eginition Hospital approved this retrospective study. The ethical guidelines of the 1964 Declaration of Helsinki were applied. All patients or next-of-kin caregivers in cases of significant cognitive decline provided written informed consent.

### Imaging data acquisition

Brain MRI was performed in all subjects on a 3 T Achieva TX Philips MRI scanner (Philips, Best, the Netherlands). The acquisition protocol included a high-resolution 3D T1-weighted Turbo Field Echo sequence with an inversion recovery excitation pulse in order to optimize gray/white matter contrast (repetition time (TR): 9.9 ms; time echo (TE): 3.7 ms; flip angle: 70; voxel size 1 × 1x1 mm; inversion time (TI): 1250 ms; sagittal orientation).

### Imaging data processing

All linear, planimetric and volumetric measurements were performed on a 3D T1-weighted sequence (see 2.3.).

The following linear measurements were performed: (a) maximal anterior–posterior (A-P) midbrain distance: M_d_; (b) maximal A-P pons distance (drawn manually, parallel to the chiasmatico-commissural (C–C) line, on the mid-sagittal plane, as described previously): P_d_ [29]; (c) maximal widths of the middle cerebellar peduncles (MCP) bilaterally (measured on a parasagittal plane); (d) bilateral superior cerebellar peduncle (SCP) widths (measured on a coronal level, as described previously) [18]; (e) maximal third ventricle and; (f) maximal frontal horns width (measured on an axial plane). [30]

In addition to these measurements, the following composite linear-based indices were calculated: (a) mean MCP width: MCP; (b) mean SCP width: SCP_d_; (c) mean SCP width x midbrain A-P distance: SCP_d_ x M_d_; (d) P/M = $\frac{Pd}{Md}$; (e) P/M 2.0 = $\frac{Pd}{Md} x \frac{3rd ventricle width}{Frontal horns width}$; (f) MRPI linear (*MRPI*_*d*_$=\frac{Pd x MCP}{Md x SCPd}$); (g) MRPI 2.0 linear (MRPI_d_ 2.0 = $\frac{Pd x MCP}{Md x SCPd} x \frac{3rd ventricle width}{Frontal horns width}$. All linear measurements were drawn using Radiant Dicom Viewer®, version 2024.1 (2009–2024, Medixant).

The following planimetric (i.e., surface-based) measurements were performed: a) midbrain surface: M_s_, and (b) pons surface: P_s_. These were traced manually on the mid-sagittal level, as described previously [29]. In addition to these measurements, the following composite surface-based indices were calculated: a) P/M_s_ = $\frac{Ps}{Ms}$; b) P/M_s_ 2.0 ratio = $\frac{Ps}{Ms} x \frac{3rd ventricle width}{Frontal horns width}$; (c) Classical MRPI (MRPI_s_$=\frac{Ps x MCP}{Ms x SCPd}$) [18]; (d) MRPI_s_ 2.0= $\frac{Ps x MCP}{Ms x SCPs} x \frac{3rd ventricle width}{Frontal horns width}$ [30]. All planimetric measurements were drawn using Radiant Dicom Viewer®, version 2024.1 (2009–2024, Medixant).

The following volumetric measurements were performed: (a) midbrain: M_v_; (b) pons: P_v_; (c) SCP and; (d) brainstem volume: B_v_. These volumes were measured by the brainstem pipeline of Freesurfer (version 7.4.1.; https://surfer.nmr.mgh.harvard.edu) [31, 32]. In addition to these measurements, the following composite volume-based indices were calculated: (a) P/B_v_ = $\frac{Pv}{Bv}$; (b) M/B = $\frac{Mv}{Bv}$; c) P/M_v_= $\frac{Pv}{Mv}$ MRPI_v_$=\frac{Pv}{Mv x SCPv}$) [18].

### Statistical analysis

Comparison of demographic and clinical data among study groups was performed by Pearson *x* [2] test, analysis of variance (ANOVA) or Kruskal–Wallis test, as appropriate. All MRI morphometric measurements were tested for normality of distribution and homogeneity of variances by skewness and kurtosis values, as well as the Kolmogorov–Smirnov test, with Lilliefors significance correction. In cases of non-normality, log-transformation was performed in an effort to restore normality of distribution.

Comparison of MRI morphometric measurements among study groups was performed by analysis of covariance, with age and disease duration as covariates and diagnosis as fixed factor in cases of normality, followed by pair-wise comparisons with Bonferroni adjustment for multiple comparisons. In cases of non-normality, Quade non-parametric analysis of covariance was applied.

Receiver Operating Characteristic (ROC) curve analysis was applied to determine the discriminative value of each MRI measurement. Optimal cut-off values were extracted, based on the maximal combined sensitivity and specificity criterion. Areas under the curve (AUC) with 95% confidence intervals (95% CI), p values, sensitivity (%), specificity (%), positive and negative Likelihood Ratios and Youden indices.

The discriminative value of each imaging marker, based on the ROC curce analysis was performed for: (a) all PSP patients; (b) Probable vs. possible/suggestive PSP patients; (c) PSP-RS vs. PSP variants (i.e., non-PSP-RS); (d) Sub-cohorts of PSP patients based on disease duration (i.e., ≤ 48 months vs. ≤ 36 months vs. ≤ 24 months).

In addition, imaging markers were calculated for each PSP phenotype.

Spearman’s rho (*ρ*) was applied to examine correlations between distance-, surface- and volume-based measurements, where available: (a) midbrain (M_d_ vs. M_s_ vs. M_v_ vs. M/B); (b) SCP (SCP_d_ vs. SCP_v_) and (c) P/M ratios (P/M_d_ vs. P/M_s_ vs. P/M_v_).

Additional correlation analyses based on Spearman’s ρ were performed in order to examine correlations of the midbrain surface (as the most potent MRI marker; see 3. Results) and PSPRS total score and domain sub-scores.

Analyses were performed by IBM SPSS Statistics® version 29.0.0.0 (SPSS Inc., Chicago, IL, 2022) and Medcalc®, version 14.8.1 (MedCalc Software, Ostend, Belgium).

## Results

### Clinical–demographic data

A total of 244 subjects were included in this study, comprising 38 CBD patients, 45 MSA patients, 80 PSP patients, 36 PD patients and 45 control subjects. Study groups differed in mean age (p = 0.005), whereas median disease duration varied from 24 to 36 months among groups. 84.9% of the patients (169/199) fulfilled criteria for established or probable diagnosis, whereas 15.1% of patients fulfilled criteria for possible/suggestive diagnosis (30/199). 78.9% (157/199) of patients had a disease duration of ≤ 48 months, 70.4% (140/199) had a disease duration of < 36 months and 52.3% (104/199) patients had a disease duration of ≤ 24 months. Differences were present in neuropsychological scores among patient groups as expected, with CBD patients performing poorer overall in memory, frontal-executive and visuospatial tests, PSP patients exhibiting pronounced frontal-executive deficits, whereas PD and MSA groups exhibited minimal neuropsychological deficits (Table 1).

**Table 1:** Demographic, clinical and neuropsychological characteristics of study subgroups

	CBD	MSA	PSP	PD	Controls	All	p value
	*n* = *38*	*n* = *45*	*n* = *80*	*n* = *36*	*n* = *45*	*N* = *244*	
Sex (*m/f*)	17/21	26/19	41/39	21/15	18/27	123/121	0.361^†^
Age (*y*)	66.0 (8.0)	62.9 (6.5)	67.9 (6.1)	65.3 (9.1)	64.5 (7.3)	65.8 (7.4)	0.005^‡^
Disease duration (*m*)	24 (18–36)	36 (24–48)	36 (24–48)	36 (12–84)	NA	36 (24–48)	< 0.001^≠^
Level of diagnostic certainty							
Established	NA	31	NA	36	NA	67	
Probable	31	14	57	(-)	NA	102	
Possible	7	(-)	18	NA	NA	25	
Suggestive	NA	NA	5	NA	NA	5	
Disease duration							
≤ 48 months	36	36	62	23	NA	157	
≤ 36 months	33	30	55	22	NA	140	
≤ 24 months	25	19	33	27	NA	104	
UPDRS III	17.5 (8–27)	17.5 (4.5–32.5)	19.5 (13–32)	23 (10–32)	NA	19 (10–31)	< 0.001^≠^
MMSE (*0–30*)	22.5 (17–25.5)	28 (26–29)	26 (23–28)	28.5 (26–30)	29 (27–30)	26 (23–29)	< 0.001^≠^
FAB (*0–18*)	9 (7–12)	14 (12–16)	10 (7–13)	16 (11–17)	16 (13–18)	12 (9–15)	< 0.001^≠^
5 WR (im) (*0–5*)	5 (4–5)	5 (5–5)	5 (5–5)	5 (5–5)	5 (5–5)	5 (5–5)	< 0.001^≠^
5 WR (del) (*0–5*)	5 (4–5)	5 (5–5)	5 (4–5)	5 (5–5)	5 (5–5)	5 (4–5)	< 0.001^≠^
Clox 1 (*0–15*)	4,5 (1–9)	11 (6,5–12)	8 (5–11)	11 (10–12)	14 (12–15)	9 (6–12)	0.004^≠^
Clox 2 (*0–15*)	8 (3–12)	13 (12–14)	11 (8–13)	12 (10–14)	15 (13,5–15)	12 (9–14)	0.014^≠^

All data are presented as mean (SD) or median (25th quartile- 75th quartile) as appropriate; UPDRS: Unified Parkinson’s disease Rating Scale III; MMSE: Mini Mental State Examination: FAB: Frontal Assessment Battery; 5 WR (im): 5-word recall test immediate; 5 WR (del): 5-word recall test delayed; Clox 1: 15-point clock drawing test (spontenous); Clox 2: 15-point clock drawing test (copy); †: *x*^2^ test; ‡: ANOVA; ≠ : Kruskal–Wallis test

### MRI data

All distance-, surface- and volume-based MRI markers differed significantly among groups. PSP patients exhibited significantly lower midbrain and SPC-based MRI marker values and higher P/M and MRPI-based values, irrespective of measurement modality, as expected (Table 2).

**Table 2:** Distance-based, surface-based and volume-based morphometric MRI markers’ data across study groups

	CBD	MSA	PSP	PD	Controls	p value
	*n* = *38*	*n* = *45*	*n* = *80*	*n* = *36*	*n* = *45*	
*Distance-based markers*						
M_d_ (*mm*)	10.5 (9.6–11.3)	11.4 (10.5–11.9)	9.0 (8.2–9.9)	11.4 (10.6–12.4)	12.3 (11.4–13.0)	< 0.001§
SCP_d_ (*mm*)	3.0 (0.4)	3.0 (0.6)	2.6 (0.7)	3.6 (0.3)	3.2 (0.4)	< 0.001*
SCP_d_ x M_d_ (*mm*^*2*^)	31.0 (6.5)	34.1 (7.7)	23.4 (7.9)	41.1 (5.8)	38.4 (5.4)	< 0.001*
P/M_d_	2.1 (2.0–2.3)	1.8 (1.6–2.0)	2.3 (2.2–2.6)	2.0 (1.9–2.2)	1.9 (1.8–2.0)	< 0.001§
P/M_d_ 2.0	1.05 (0.77–1.31)	0.54 (0.42–0.66)	1.50 (1.08–2.13)	0.86 (0.73–1.03)	0.60 (0.44–0.79)	< 0.001§
MRPI_d_	6.3 (5.5–6.9)	4.0 (3.3–4.7)	7.5 (6.0–9.0)	5.3 (4.7–5.7)	5.4 (5.0–6.0)	< 0.001§
MRPI_d_ 2.0	1.4 (1.2–1.7)	0.7 (0.5–0.9)	2.0 (1.4–2.8)	1.1 (0.9–1.3)	0.9 (0.7–1.2)	< 0.001#
*Surface-based markers*						
M_s_ (*mm*^*2*^)	118.2 (24.8)	143.5 (22.9)	86.6 (21.1)	139.9 (27.8)	152.8 (21.8)	< 0.001*
P/M_s_	4.3 (3.7–5.2)	3.0 (2.6–3.6)	5.7 (4.8–6.9)	4.1 (3.6–4.6)	3.7 (3.2–3.8)	< 0.001§
P/M_s_ 2.0	1.05 (0.77–1.31)	0.54 (0.42–0.66)	1.50 (1.08–2.13)	0.86 (0.73–1.03)	0.60 (0.44–0.79)	< 0.001#
MRPI_s_	13.0 (10.6–15.7)	6.1 (4.9–8.6)	17.5 (13.8–24.3)	10.3 (9.2–11.6)	10.4 (9.3–11.6)	< 0.001§
MRPI_s_ 2.0	2.9 (2.3–3.6)	1.2 (0.8–1.7)	4.7 (3.1–7.1)	2.2 (1.9–2.6)	1.7 (1.2–2.3)	< 0.001#
*Volume-based markers*						
M_v_ (*mm*^*3*^)	5514 (746)	5622 (663)	4926 (605)	6149 (635)	5812 (577)	< 0.001*
M/B	0.23 (0.22–0.24)	0.27 (0.24–0.29)	0.23 (0.22–0.24)	0.23 (0.23–0.24)	0.23 (0.23–0.24)	< 0.001§
SPC_v_ (*mm*^*3*^)	248.6 (60.3)	219.0 (69.5)	221.4 (67.3)	278.2 (62.8)	261.3 (54.3)	< 0.001*
P/M_v_	2.49 (2.40–2.61)	1.99 (1.67–2.36)	2.56 (2.39–2.67)	2.48 (2.38–2.53)	2.48 (2.40–2.57)	< 0.001§
MRPI_v_ (× 100)	1.03 (0.60–1.16)	0.97 (0.83–1.14)	1.17 (1.00–1.49)	0.93 (0.80–1.07)	0.99 (0.84–1.09)	< 0.001§

All data are presented as mean (SD) or median (25th quartile- 75th quartile) as appropriate; §: Quade Nonparametric ANCOVA; *: ANCOVA, with age and disease duration as covariates; #: ANCOVA, with age and disease duration as covariates, after logarithmic transformation of data

### Comparison of diagnostic accuracy of distance, surface and volume-based MRI markers

Among distance-based markers, M_d_ provided the maximal diagnostic accuracy (AUC = 0.889; 82.5% sensitivity; 87.3% specificity) in discriminating PSP from non-PSP subjects. This was the only distance-based marker to provide combined sensitivity and specificity of > 80%. P/M_d_, P/M_d_ 2.0 and MRPI_d_ 2.0 produced AUCs > 0.850, with combined sensitivity and specificity of > 75%.

M_s_ provided the maximal diagnostic accuracy among surface-based markers for discriminating PSP from non-PSP subjects (AUC = 0.937; 88.8% sensitivity; 84.2% specificity). All surface-based markers produced AUC values > 0.870, with combined sensitivity/specificity > 80%, with the exception of P/Ms 2.0, which resulted in suboptimal specificity (70.7%) in the differentiation of PSP vs. non-PSP subjects.

Among volume-based markers, Mv provided optimal diagnostic accuracy (AUC = 0.819; 70% sensitivity; 83.5% specificity). All other MRI-based markers provided poor sensitivity (42.5%-52.5%).

**Fig. 1:**
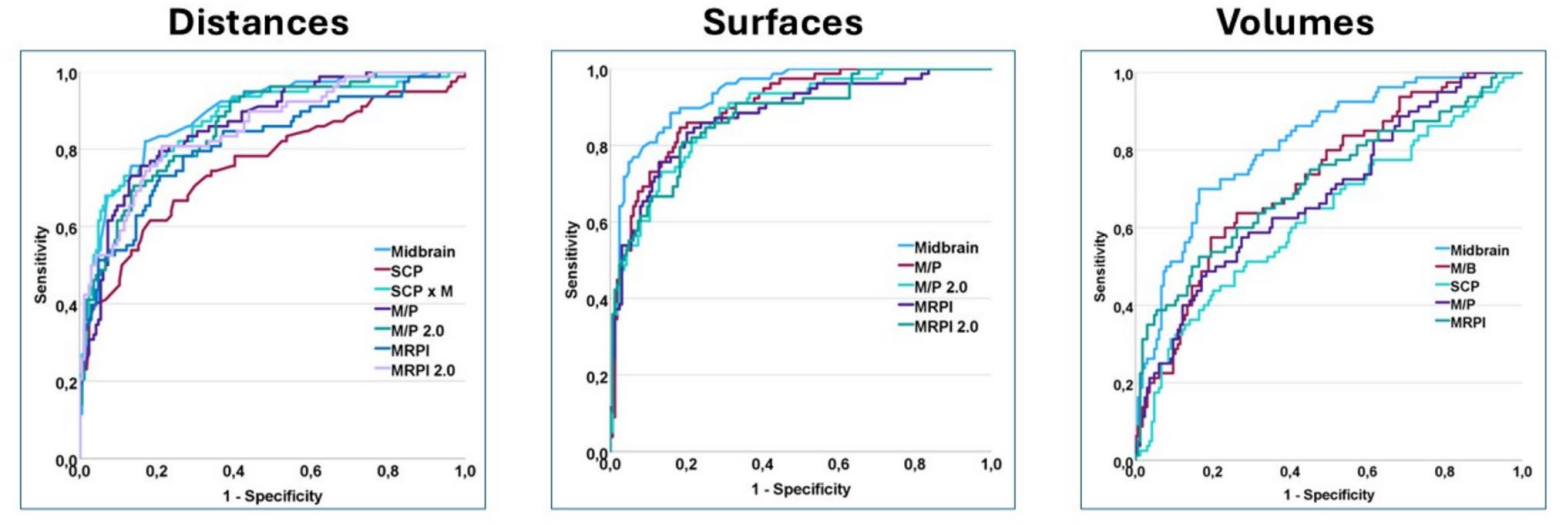
ROC curves of MRI markers based on modality: **a** distance-based MRI markers; **b** surface-based MRI markers; **c** volume-based MRI markers

In all three measurement modality categories (distance-, surface-, volume-based), simple midbrain measurements (i.e., M_d_, M_s_, M_v_) produced the highest AUC values and optimal combined sensitivity/specificity compared to SCP measurements and composite markers (i.e., P/M and MRPI). In addition, surface-based markers in general were superior to distance- and volume-based markers, with volume-based markers in particular resulting in poor diagnostic accuracy (Table 3, Fig. 1).

**Table 3:** Diagnostic accuracy of distance-based, surface-based and volume-based MRI markers in the differentiation of PSP from all other study groups combined (i.e., PSP vs. non-PSP subjects)

	Cut-off	AUC (95% CI)	p value	Sens. (%)	Spec. (%)	+ LR	-LR	Youden Index
*Distance-based markers*								
M_d_ (*mm*)	≤ 10.2	0.889 (0.843–0.926)	< 0.001	82.5	87.3	4.67	0.21	0.65 (0.54–0.73)
SCP_d_ (*mm*)	≤ 2.70	0.767 (0.709–0.819)	< 0.001	62.5	81.7	3.42	0.46	0.44 (0.32–0.53)
SCP_d_ x M_d_ (*mm*^*2*^)	≤ 27.3	0.878 (0.830–0.916)	< 0.001	73.8	88.4	6.37	0.30	0.62 (0.52–0.70)
P/M_d_	> 2.19	0.866 (0.817–0.906)	< 0.001	76.3	84.2	4.81	0.28	0.60 (0.48–0.69)
P/M_d_ 2.0	> 0.52	0.859 (0.808–0.900)	< 0.001	70.5	86.0	5.03	0.34	0.57 (0.46–0.65)
MRPI_d_	> 6.14	0.819 (0.765–0.865)	< 0.001	73.8	78.7	3.46	0.33	0.52 (0.37–0.60)
MRPI_d_ 2.0	> 1.35	0.851 (0.800–0.894)	< 0.001	80.8	78.1	3.68	0.25	0.59 (0.47–0.68)
*Surface-based markers*								
M_s_ (*mm*^*2*^)	≤ 115.1	0.937 (0.899–0.964)	< 0.001	88.8	84.2	6.06	0.17	0.73 (0.63–0.79)
P/M_s_	> 4.55	0.903 (0.859–0.937)	< 0.001	83.8	82.3	4.74	0.20	0.66 (0.54–0.73)
P/M_s_ 2.0	> 0.93	0.881 (0.834–0.919)	< 0.001	89.7	70.7	3.07	0.15	0.61 (0.48–0.68)
MRPI_s_	> 12.6	0.881 (0.833–0.918)	< 0.001	83.8	79.9	4.04	0.23	0.64 (0.52–0.71)
MRPI_s_ 2.0	> 2.93	0.874 (0.826–0.707)	< 0.001	80.8	81.1	4.27	0.24	0.62 (0.50–0.71)
*Volume-based markers*								
M_v_ (*mm*^*3*^)	≤ 5155	0.819 (0.765–0.865)	< 0.001	70	83.5	4.25	0.36	0.54 (0.42–0.64)
M/B	≤ 0.22	0.709 (0.648–0.766)	< 0.001	42.5	85.4	2.49	0.69	0.28 (0.18–0.36)
SPC_v_ (*mm*^*3*^)	≤ 198	0.632 (0.568–0.693)	< 0.001	43.8	79.9	2.49	0.64	0.24 (0.11–0.32)
P/M_v_	> 2.58	0.684 (0.621–0.741)	< 0.001	47.5	82.9	2.48	0.95	0.30 (0.18–0.40)
MRPI_v_ (× 100)	> 0.012	0.716 (0.655–0.771)	< 0.001	52.5	82.3	2.97	0.58	0.35 (0.23–0.43)

AUC (95% CI): Area Under the Curve (95% Confidence Interval); *Sens*. Sensitivity, *Spec*. Specificity; + *LR* positive Likelihood Ratio, −*LR* negative Likelihood Ratio

### Diagnostic accuracy of MRI markers in probable vs. possible PSP

MRI markers provided higher diagnostic accuracy in differentiating probable PSP patients compared to possible PSP patients from non-PSP subjects. M_s_ provided the highest diagnostic accuracy for discriminating both probable PSP (AUC = 0.953; 87.7% sensitivity; 93.4% specificity) and possible PSP patients (AUC = 0.900; 95.7% sensitivity; 71.4% specificity) from non-PSP subjects. Combined sensitivity/specificity was > 80% for the five optimal MRI markers in differentiating probable PSP patients from non-PSP subjects, whereas none of the MRI markers differentiated possible PSP subjects from non-PSP subjects with combined sensitivity/specificity of > 75% (Table 4, Supplementary Fig. 1).

**Table 4:** Diagnostic accuracy of MRI markers in the discrimination of: (a) probable PSP vs. non-PSP subjects; (b) possible/suggestive PSP vs. non-PSP subjects

	Cut-off	AUC (95% CI)	p value	Sens. (%)	Spec (%)	+ LR	-LR	Youden Index
*Probable PSP*								
M_s_ (*mm*^*2*^)	≤ 104	0.953 (0.916–0977)	< 0.001	87.7	93.4	14.4	0.13	0.82 (0.70–0.89)
P/M_s_	> 5.07	0.936 (0.895–0.964)	< 0.001	82.5	91.5	9.7	0.19	0.74 (0.63–0.79)
P/M_s_ 2.0	> 1.16	0.927 (0.884–0.958)	< 0.001	85.5	86.6	6.4	0.17	0.72 (0.60–0.80)
MRPI_s_ 2.0	> 2.99	0.922 (0.879–0.954)	< 0.001	89.1	81.7	4.9	0.13	0.71 (0.59–0.77)
MRPI_s_	> 13.8	0.921 (0.878–0.953)	< 0.001	87.7	87.2	9.5	0.14	0.75 (0.63–0.83)
*Possible/suggestive PSP*								
M_s_ (*mm*^*2*^)	≤ 123	0.900 (0.847–0.939)	< 0.001	95.7	71.4	3.3	0.06	0.67 (0.55–0.72)
M_d_ *(mm)*	≤ 10.2	0.838 (0.778–0.888)	< 0.001	73.9	82.3	4.2	0.32	0.56 (0.33–0.69)
P/M_s_	> 4.15	0.821 (0.759–0.874)	< 0.001	82.6	69.5	2.7	0.25	0.52 (0.32–0.62)
SCP_d_ x M_d_ *(mm*^*2*^*)*	≤ 34.2	0.803 (0.738–0.857)	< 0.001	91.3	60.4	2.3	0.14	0.52 (0.29–0.61)
MRPI_s_	> 12.4	0.779 (0.713–0.837)	< 0.001	73.9	78.1	3.4	0.33	0.52 (0.32–0.67)

For each analysis, only the five optimal MRI markers are presented, based on AUC values. *AUC (95% CI)* Area Under the Curve (95% Confidence Interval), *Sens*. Sensitivity, *Spec*. Specificity; + *LR* positive Likelihood Ratio, -*LR* negative Likelihood Ratio

**Table 7:** Diagnostic accuracy of MRI markers in the discrimination of: (a) PSP-RS vs. non-PSP subjects; (b) PSP variants vs. non-PSP subjects

	Cut-off	AUC (95% CI)	p value	Sens. (%)	Spec (%)	+ LR	-LR	Youden Index
*PSP-RS*								
M_s_ (*mm*^*3*^)	≤ 102	0955 (0.918–0.979)	< 0.001	86.0	95.1	17.6	0.15	0.81 (0.69–0.88)
P/M_s_	> 5.12	0.937 (0.896–0.966)	< 0.001	82.0	92.7	11.2	0.19	0.75 (0.60–0.81)
MRPI_s_	> 13.8	0.931 (0.889–0.961)	< 0.001	92.0	87.2	7.2	0.09	0.79 (0.67–0.87)
MRPI_s_ 2.0	> 2.99	0.924 (0.880–0.956)	< 0.001	89.8	81.7	4.9	0.12	0.72 (0.59–0.79)
P/M_s_ 2.0	> 1.34	0.924 (0.879–0.956)	< 0.001	77.6	92.1	9.8	0.24	0.70 (0.57–0.78)
*PSP variants*								
M_s_ (*mm*^*3*^)	≤ 115	0.908 (0.858–0.944)	< 0.001	83.3	84.2	5.3	0.20	0.68 (0.54–0.77)
M_d_	≤ 10.2	0.853 (0.795–0.900)	< 0.001	76.7	82.3	4.3	0.28	0.59 (0.42–0.72)
P/M_s_	> 4.06	0.845 (0.787–0.893)	< 0.001	86.7	67.1	2.6	0.20	0.54 (0.35–0.61)
SCP_d_ x M_d_	≤ 34.2	0.824 (0.763–0.875)	< 0.001	93.3	60.4	2.4	0.11	0.54 (0.37–0.63)
MRPI_s_	> 12.4	0.796 (0.733–0.850)	< 0.001	73.3	78.1	3.3	0.34	0.51 (0.32–0.64)

For each analysis, only the five optimal MRI markers are presented, based on AUC values. *AUC (95% CI)* Area Under the Curve (95% Confidence Interval), *Sens*. Sensitivity, *Spec*. Specificity,  + *LR* positive Likelihood Ratio, -*LR* negative Likelihood Ratio (Table 7, Supplementary Fig. 2)

### Diagnostic accuracy of MRI markers in PSP-RS vs. PSP variants

Multiple MRI markers differentiated PSP-RS from non-PSP subjects with > 80% sensitivity/specificity. M_s_ was the optimal MRI marker for PSP-RS vs. non-PSP subjects’ differentiation (AUC = 0.955; 86.0% sensitivity; 95.1% specificity). M_s_ was the only marker which differentiated PSP variants from non-PSP subjects with > 80% sensitivity/specificity (AUC = 0.908; 83.3% sensitivity; 84.2% specificity).

### Diagnostic accuracy of MRI markers in PSP patients stratified by disease duration

**Fig. 2:**
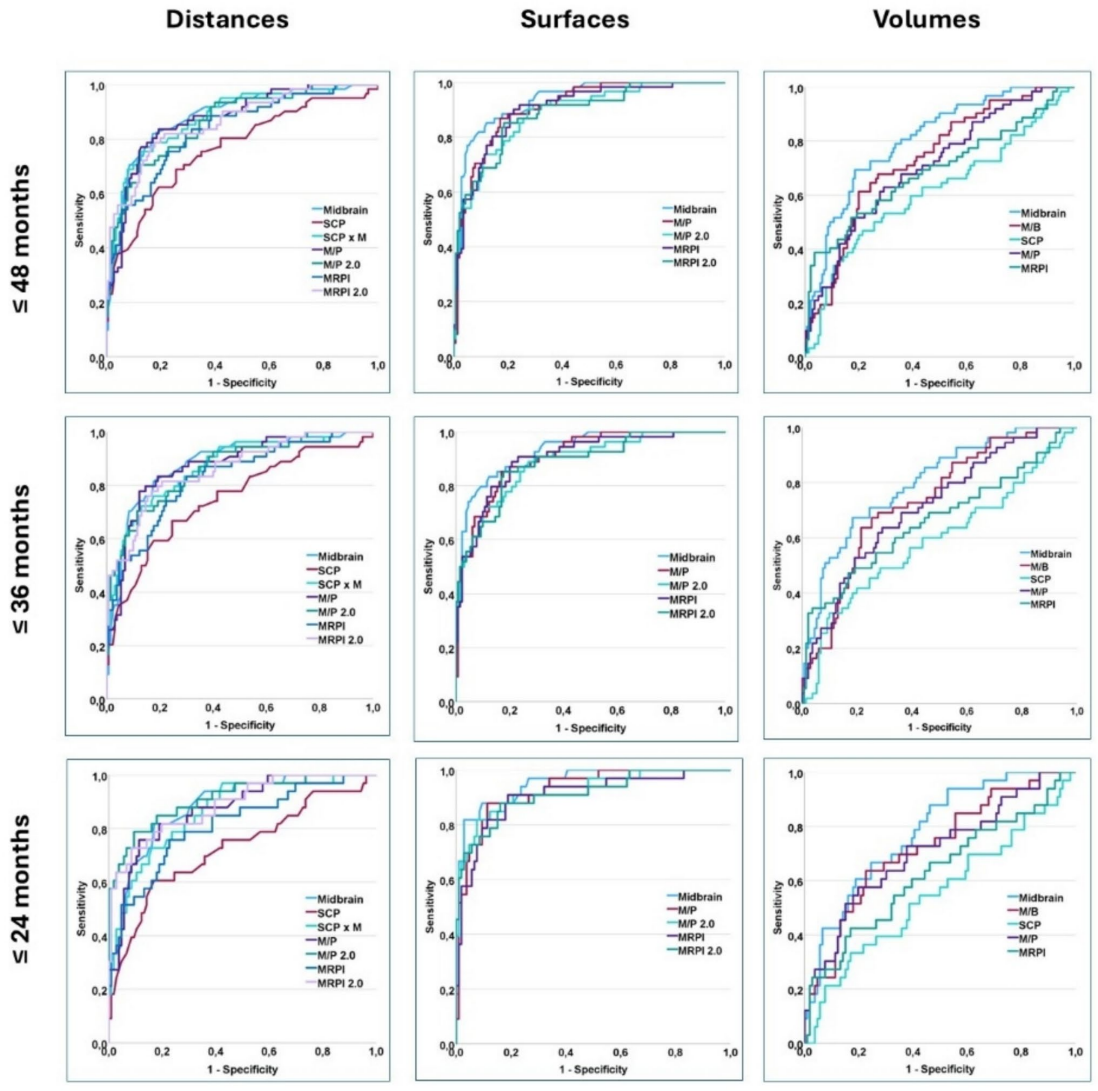
ROC curves of MRI markers based on disease duration

Stratifying patients based on disease duration (i.e., ≤ 48 months vs. ≤ 36 months vs. ≤ 24 months) did not significantly alter the diagnostic accuracy of MRI marker in differentiating PSP from non-PSP subjects. Irrespective of disease duration, M_s_, P/M_s_, MRPI_s_ and MRPI_s_ 2.0 were among the five optimal imaging markers, supporting the overall superiority of surface-based MRI markers in diagnosing PSP in early as well as later disease stages. Interestingly, optimal cut-off values were stable for MRPI_s_ and MRPI_s_ 2.0 across the three disease-duration stratifications. M_s_ and P/M_s_ optimal cut-offs varied slightly in the ≤ 24-month cohort compared to the ≤ 36-month and ≤ 48-month cohorts (Table 5, Fig. 2).

**Table 5:** Diagnostic accuracy of MRI markers in the discrimination of all PSP vs. non-PSP subjects stratified by disease duration: (a) Patients with disease duration ≤ 48 months; (b) Patients with disease duration ≤ 36 months; (c) Patients with disease duration ≤ 24 months

	Cut-off	AUC (95% CI)	p value	Sens. (%)	Spec (%)	+ LR	-LR	Youden Index
*Disease duration* ≤ *48 months*								
M_s_ (*mm*^*2*^)	≤ 110	0.934 (0.890–0.964)	< 0.001	85.5	87.9	7.0	0.17	0.73 (0.62–0.81)
P/M_s_	> 4.55	0.910 (0.861–0.945)	< 0.001	87.1	82.8	5.1	0.16	0.72 (0.60–0.79)
MRPI_s_	> 12.4	0.903 (0.854–0.940)	< 0.001	90.3	77.9	4.1	0.12	0.68 (0.53–0.76)
MRPI_s_ 2.0	> 2.93	0.887 (0.835–0.927)	< 0.001	85.3	81.4	4.6	0.18	0.67 (0.54–0.76)
M_d_ (*mm*)	≤ 10.2	0.883 (0.830–0.924)	< 0.001	82.3	82.1	4.6	0.22	0.64 (0.50–0.74)
*Disease duration* ≤ *36 months*								
M_s_ (*mm*^*2*^)	≤ 110	0.930 (0.883–0.962)	< 0.001	83.6	87.7	6.8	0.19	0.71 (0.58–0.80)
P/M_s_	> 4.55	0.909 (0.857–0.946)	< 0.001	85.5	83.1	5.1	0.18	0.69 (0.55–0.78)
MRPI_s_	> 12.4	0.903 (0.851–0.942)	< 0.001	89.1	79.2	4.3	0.14	0.68 (0.54–0.77)
P/M_s_ 2.0	> 0.94	0.888 (0.834–0.930)	< 0.001	88.9	73.1	3.3	0.15	0.62 (0.47–0.70)
MRPI_s_ 2.0	> 2.93	0.886 (0.832–0.928)	< 0.001	85.2	82.3	4.8	0.18	0.67 (0.54–0.77)
*Disease duration* ≤ *24 months*								
M_s_ (*mm*^*3*^)	≤ 101	0.952 (0.902–0.981)	< 0.001	81.8	97.2	28.9	0.19	0.79 (0.64–0.88)
P/M_s_	> 4.7	0.928 (0.872–0.965)	< 0.001	87.9	88.7	7.8	0.14	0.77 (0.60–0.87)
P/M_s_ 2.0	> 1.18	0.928 (0.872–0.965)	< 0.001	81.8	92.5	10.8	0.20	0.74 (0.56–0.84)
MRPI_s_ 2.0	> 2.93	0.913 (0.853–0.954)	< 0.001	87.9	84.0	5.5	0.14	0.72 (0.54–0.82)
MRPI_s_	> 12.4	0.908 (0.847–0.950)	< 0.001	90.9	81.1	4.8	0.44	0.72 (0.54–0.81)

For each analysis, only the five optimal MRI markers are presented, based on AUC values. *AUC (95% CI)* Area Under the Curve (95% Confidence Interval), *Sens.* Sensitivity, *Spec.* Specificity,  + *LR* positive Likelihood Ratio, -*LR* negative Likelihood Ratio

### MRI imaging markers in different PSP phenotypes

PSP variants exhibited significant differences in most imaging markers compared to PSP-RS, indicating less pronounced midbrain and SCP atrophy, thus resulting in greater P/M and MRPI-based composite markers’ values. Among the different PSP variants, PSP-F exhibited midbrain atrophy numerically comparable to PSP-RS. However, due to the small samples of sub-cohorts, statistical analysis could not be performed on an individual PSP variant basis (Table 6, Supplementary Fig. 3).

**Table 6:** Demographic, clinical, neuropsychological characteristics and morphometric MRI markers’ data of study subgroups

	PSP-RS *n* = *50*	PSP variants								
		All *n* = *30*	PSP-PGF *n* = *1*	PSP-P *n* = *1*	PSP-OM *n* = *1*	PSP-PI *n* = *3*	PSP-F *n* = *7*	PSP-SL *n* = *5*	PSP-CBS *n* = *12*	p value
*Demographic/clinical data*										
Sex (m/f)	27/23	14/16	1/0	0/1	0/1	1/2	5/2	4/1	3/9	0.207^†^
Age (y)	67.0 (6.6)	69.1 (4.9)	57 (-)	74 (-)	77 (-)	67.3 (5.9)	66.4 (3.0)	70.4 (3.7)	70.3 (4.1)	0.131^‡^
Disease duration (m)	27 (24–36)	36 (24–60)	60 (-)	60 (-)	8 (-)	36 (18–42)	60 (36–60)	36 (36–60)	36 (19–54)	0.029^≠^
*PSP Rating Scale*										
Total	32.8 (10.8)	25.5 (10.1)	11 (-)	37 (-)	14 (-)	29.7 (12.1)	31.8 (8.3)	17.0 (6.9)	26.1 (8.9)	0.0071^‡^
Cognitive	3.8 (2.6)	3.6 (2.6)	1.0 (-)	1.0 (-)	3.0 (-)	5.7 (4.6)	5.0 (2.9)	2.8 (1.7)	3.2 (1.9)	0.2871^‡^
Bulbar	2.5 (1.3)	1.7 (1.1)	0.0 (-)	3.0 (-)	0.0 (-)	1.7 (1.5)	1.4 (0.9)	1.8 (1.0)	2.0 (1.1)	0.0081^‡^
Eyes	6.7 (2.5)	3.4 (3.0)	0.0 (-)	1.0 (-)	3.0 (-)	1.7 (1.2)	5.8 (3.0)	2.5 (1.0)	3.8 (3.7)	< 0.00011^‡^
Limbs	3.2 (2.1)	4.4 (2.9)	1.0 (-)	9.0 (-)	5.0 (-)	4.3 (2.9)	3.4 (1.8)	3.0 (1.6)	5.3 (3.7)	0.0811^‡^
Gait	9.9 (4.4)	6.5 (4.0)	6.0 (-)	14.0 (-)	2.0 (-)	7.3 (4.2)	8.1 (0.2)	4.3 (3.0)	6.1 (4.9)	0.0021^‡^
*Distance-based markers*										
M_d_	8.9 (8.0–9.6)	9.4 (8.4–10.2)	10.2 (-)	10.2 (-)	12.0 (-)	10.2 (8.9–11.6)	8.6 (8.2–9.3)	9.5 (8.1–9.8)	9.4 (8.4–10.6)	0.137§
SCP_d_	2.5 (0.7)	2.74 (0.72)	2.1 (-)	3.0 (-)	2.9 (-)	2.5 (0.7)	2.6 (0.7)	2.3 (0.8)	3.1 (0.7)	0.091*
SCP x M	22.0 (7.2)	25.8 (8.6)	21.4 (-)	30.1 (-)	34.2 (-)	24.7 (4.3)	22.8 (7.3)	20.8 (8.6)	29.2 (9.7)	0.049*
P/M_d_	2.37 (2.28–2.59)	2.23 (2.09–2.43)	2.01 (-)	2.11 (-)	1.89 (-)	1.95 (1.90–2.37)	2.43 (2.30–2.57)	2.61 (2.34–2.69)	2.19 (2.10–2.27)	0.010§
P/M_d_ 2.0	0.72 (0.53–0.83)	0.55 (0.45–0.61)	0.49 (-)	0.44 (-)	0.59 (-)	0.45 (0.38–0.60)	0.76 (0.58–0.77)	0.59 (0.56–0.68)	0.46 (0.40–0.53)	0.001§
MRPI_d_	7.87 (6.33–9.75)	6.52 (5.36–7.65)	5.07 (-)	6.68 (-)	6.31 (-)	6.05 (4.81–10.2)	7.72 (5.76–9.11)	7.65 (6.57–12.4)	5.67 (4.92–6.64)	0.006§
MRPI_d_ 2.0	2.29 (1.63–3.08)	1.54 (1.10–2.02)	1.23 (-)	1.38 (-)	1.97 (-)	1.54 (0.97–2.34)	2.07 (1.82–2.30)	1.65 (1.56–3.04)	1.10 (0.92–1.48)	< 0.001#
*Surface-based markers*										
M_s_	76.5 (69.0–94.0)	90.8 (73.0–112)	106.0 (-)	112.1 (-)	109.8 (-)	89.2 (84.6–93.0)	67.0 (63.0–91.6)	84.9 (73.0–104)	106.6 (86.8–123)	0.042*
P/M_s_	6.31 (5.30–6.99)	5.0 (4.22–5.78)	3.97 (-)	5.12 (-)	4.80 (-)	5.20 (4.92–5.56)	5.78 (5.08–7.83)	6.32 (4.93–8.05)	4.34 (4.02–5.07)	0.003§
P/M_s_ 2.0	1.73 (1.36–2.34)	1.21 (1.00–1.48)	0.96 (-)	1.06 (-)	1.50 (-)	1.19 (1.00–1.41)	1.51 (1.29–2.47)	1.30 (1.25–1.81)	0.98 (0.71–1.16)	< 0.001#
MRPI_s_	19.5 (15.3–25.3)	13.48 (11.99–18.04)	10.02 (-)	16.23 (-)	16.01 (-)	14.23 (12.5–27.3)	18.04 (11.9–24.4)	17.98 (13.8–38.1)	12.59 (10.4–12.9)	< 0.001§
MRPI_s_ 2.0	5.78 (3.84–8.24)	3.25 (2.52–4.80)	2.43 (-)	3.35 (-)	5.01 (-)	3.62 (2.52–6.25)	4.80 (3.79–6.66)	3.67 (3.44–8.56)	2.53 (1.64–3.09)	< 0.001#
*Volume-based markers*										
M_v_	4808 (4422–5156)	5087 (4692–5453)	5682 (-)	5086 (-)	5666 (-)	5082 (4506–5453)	4933 (4812–5087)	5404 (5132–5427)	4816 (4096–5503)	0.089*
M/B	0.22 (0.22–0.23)	0.24 (0.23–0.24)	0.24 (-)	0.21 (-)	0.24 (-)	0.23 (0.22–0.25)	0.23 (0.22–0.24)	0.24 (0.22–0.24)	0.24 (0.23–0.24)	< 0.001§
SCP_v_	221.5 (67.2)	221.2 (68.4)	249.2 (-)	231.6 (-)	358.4 (-)	209.3 (62.8)	210.5 (55.4)	267.3 (77.2)	196.6 (65.3)	0.983*
M/P_v_	2.63 (2.50–2.73)	2.40 (2.33–2.57)	2.33 (-)	2.77 (-)	2.53 (-)	2.57 (2.10–2.60)	2.39 (2.38–2.64)	2.41 (2.39–2.59)	2.36 (2.25–2.49)	< 0.001§
MRPI_v_	1.19 (1.03–1.62)	1.10 (0.94–1.35)	0.94 (-)	1.20 (-)	0.70 (-)	1.08 (0.94–1.66)	1.21 (1.08–1.35)	1.00 (0.80–1.02)	1.13 (0.99–1.59)	0.257§

All data are presented as mean (SD) or median (25th quartile- 75th quartile) as appropriate; *UPDRS* Unified Parkinson’s disease Rating Scale III, *MMSE* Mini Mental State Examination, *FAB* Frontal Assessment Battery, *5 WR (im)* 5-word recall test immediate, *5 WR (del)* 5-word recall test delayed, *Clox 1* 15-point clock drawing test (spontenous), *Clox 2* 15-point clock drawing test (copy); †: *x*^*2*^ test; ‡: ANOVA; ≠ : Kruskal–Wallis test; §: Quade Nonparametric ANCOVA; *: ANCOVA, with age and disease duration as covariates; #: ANCOVA, with age and disease duration as covariates, after logarithmic transformation of data

### Correlations between distance, surface and volume-based markers

There was significant correlation between M_d_, M_s_ and M_v_ values, with higher correlation coefficients between M_d_ and M_s_ (ρ = 0.745; *p* < 0.001), followed by M_d_ and M_v_ (ρ = 0.616; *p* < 0.001) and Ms and Mv (ρ = 0.542; *p* < 0.001). M/B also exhibited significant correlation with M_d_ (ρ = 0.204; *p* < 0.001) and M_s,_ (ρ = 0.276; *p* < 0.001) but there was no correlation between M/B and M_v_ (ρ = 0.045; *p* = 0.483). SCP_d_ and SCP_v_ also correlated significantly (ρ = 0.327; p < 0.001). Correlations were also significant between P/M ratios, with P/M_s_ and P/M_d_ exhibiting the highest correlation coefficient (ρ = 0.783; *p* < 0.001), followed by P/M_s_ and P/M_v_ (ρ = 0.509; *p* < 0.001) and P/M_d_ and P/M_v_ (ρ = 0.496; *p* < 0.001) (Supplementary Fig. 4).

### Correlations between PSPRS and imaging markers

There was significant correlation between midbrain surface and PSPRS total score (ρ = 0,375; *p* < 0.001), PSPRS eye sub-score (ρ = 0,529; *p* < 0.001), PSPRS bulbar sub-score (ρ = 0,399; *p* < 0.001), gait sub-score (ρ = 0,423; *p* < 0.001) and history subscore (ρ = 0,323; *p* < 0.001). PSPRS limb sub-score (ρ = 0,077; p = 0.446) and PSPRS cognitive subscore (ρ = 0,033; *p* = 0.742) did not exhibit significant correlations with midbrain surface (Supplementary Fig. 5).

## Discussion

Over the past two decades multiple MRI markers, based on the selective midbrain and SCP atrophy of PSP, have been introduced in an effort to increase diagnostic accuracy of this rare disease. These markers initially included simple distance, area and volume measurements but have since evolved into more complex composite markers which incorporate ≥ 2 measurements, such as the P/M ratio and the MRPI. The reported diagnostic accuracy of most of these markers is based on limited studies of small cohorts of Parkinsonian disorders, often with poor representation of the major atypical Parkinsonian disorders (i.e., MSA, PSP, CBD). In addition, these cohorts exhibit great heterogeneity regarding clinical presentation, level of diagnostic certainty and disease duration of PSP patients among others. We aimed to systematically compare the diagnostic accuracy of available PSP MRI markers and examine the possible effects of disease duration, clinical presentation and level of diagnostic certainty in the performance of these imaging markers as surrogate markers of PSP.

An important initial finding of our study was the superiority of surface-based over distance-based and volume-based MRI markers in discriminating PSP from non-PSP patients. More specifically, midbrain surface exhibited higher accuracy (AUC = 0.937) over midbrain distance (AUC = 0.889) and volume (AUC = 0.819). Limited data exists regarding the direct comparison of linear, planimetric and volumetric MRI markers. An initial study supported the superiority of midbrain area over midbrain volume in differentiating PSP from CBD patients [12]. This superiority was further supported by a subsequent study reporting high (> 90%) diagnostic accuracy of midbrain surface compared to midbrain volume and distance, in a cohort of PSP and MSA patients [13]. This finding is in accordance with a recent meta-analysis of MRI markers in parkinsonian disorders, which supported the superiority of planimetric over volumetric MRI markers [25].

Interestingly, midbrain surface was superior to all composite MRI markers (P/M, P/M 2.0, MRPI, MRPI 2.0) in differentiating PSP from non-PSP patients in our cohort. Studies directly comparing the diagnostic accuracy of midbrain area to composite MRI markers are lacking. A single study focusing on the differentiation of PSP from PD/MSA patients reported superior diagnostic accuracy of midbrain surface (90%) compared to P/M ratio (81.4%) and MRPI (82.9%) [23]. A meta-analysis on PSP imaging markers also supported the superiority of midbrain surface over composite MRI markers [25].

The development of composite MRI markers (which include pontine morphometric measurements) aimed at differentiating PSP (characterized by predominant midbrain/SCP atrophy) from MSA (characterized by predominant pons/MCP atrophy). Multiple studies however have reported a concomitant moderate degree of pontine atrophy in PSP, as opposed to PD and control subjects, where pontine atrophy is minimal or absent [5, 7, 18–20]. Thus, the inclusion of pontine measurements in P/M and MRPI may in actuality result in a decrease in diagnostic accuracy, when differentiating PSP from PD or CBD patients. This point is of great clinical importance, since it supports the use of midbrain surface over the P/M ratio and MRPI as the optimal MRI marker in differentiating PSP from other parkinsonian disorders (with the possible exception of MSA).

Volume-based measurements performed poorer than surface- and distance-based measurements in discriminating PSP patients. None of the volume-based marker reached the threshold of 80% sensitivity and specificity. The sub-optimal diagnostic accuracy of volumetric MRI markers has been previously reported [12, 13, 15]. Volumetric measurements can only be performed automatically through dedicated software in clinical practice. Thus, limitations of volumetric MRI measurements rely heavily on the imaging processing package used and cannot be generalized. For the same reason, volumetric MRI data cannot be compared across different studies. Thus, the limitations of volumetric measurements reported herein apply specifically to the Freesurfer package which was utilized in this study and cannot be generalized for other packages analyzing structural neuroimaging data. Regarding linear measurements, a non-brainstem-based linear composite MRI marker (third ventricle width/internal skull diameter) has provided high diagnostic accuracy in differentiating of PSP from PD patients, indicating that morphometric MRI markers of other brain regions may assist in the diagnosis of Parkinsonian disorders [33].

The diagnostic accuracy of MRI markers depended on the level of diagnostic certainty in our cohort, with all MRI markers performing significantly better in cases of probable PSP vs. possible/suggestive PSP patients. Multiple markers provided high (> 80%) combined sensitivity/specificity in discriminating probable PSP patients, with diagnostic accuracy decreasing significantly when the same markers were applied in PSP patients fulfilling possible or suggestive criteria for PSP. Most studies in the field have analyzed mixed (i.e., probable and possible) PSP cohorts [5, 7, 12, 15, 17–19, 21, 23]. Data on differences between possible vs. probable PSP patients are scarce. A single study on the subject agreed with the superior performance of imaging markers in cohorts of probable vs. possible PSP patients [20]. This finding implies that MRI markers may have suboptimal accuracy in cases where there is clinical uncertainty on the diagnosis, which is the context where these markers are most relevant.

MRI markers performed better in PSP-RS patients compared to PSP variants in our cohort, as previously reported [34]. Several studies have supported the suboptimal diagnostic accuracy of MRI markers in PSP-P cohorts [7, 24, 35, 36]. To this end, novel MRI markers were introduced (i.e., MRPI 2.0 and M/P 2.0). These novel MRI markers produced suboptimal diagnostic accuracy on our PSP variant cohort, which however mainly consisted of PSP-F, PSP-CBS and PSP-SL patients. This indicates that although MRPI 2.0 is a potent imaging marker for PSP-P, it does not assist in the diagnosis of other PSP variants. Of interest, midbrain surface was the only MRI marker which provided high diagnostic accuracy in discriminating PSP variants and could be utilized in this context.

When analyzing MRI data among different PSP phenotypes, differences emerged, with PSP-F in our cohort exhibiting midbrain atrophy numerically comparable to PSP-RS. Previous studies have supported this finding, indicating that among PSP variants, PSP-F may present the greatest imaging similarities to PSP-RS [35]. However, due to the small number of patients per PSP phenotype, safe conclusions on this subject cannot be reached. Further studies are needed to replicate these findings.

An additional finding of this study was the effect of disease duration on the diagnostic accuracy of MRI markers. Importantly, there were no significant differences in the performance of these markers when patients were stratified based on disease duration. Even when applied in PSP patients with a disease duration of ≤ 24 months, all MRI markers retained their diagnostic accuracy. In addition, there were no significant differences regarding optimal cut-off values of MRI markers and which MRI markers were optimal for PSP diagnosis when cohorts of different disease duration were analyzed. Several studies support this finding. A study including MRI scans performed prior to disease onset in a small cohort of PSP patients indicated that midbrain atrophy precedes clinical manifestations [37]. Another study indicated that baseline MRI morphometric characteristics can can identify patients with unclassified Parkinsonism who develop PSP at follow-up [38]. These findings are clinically relevant, since they imply that morphometric changes present early in the disease course of PSP patients, and thus MRI markers can be applied in PSP patients in early stages of their disease course.

Lastly, we looked at possible correlations between midbrain surface (as the optimal MRI marker for PSP) and clinical characteristics of PSP patients, based on PSPRS total score and domain sub-scores. Midbrain surface correlated with total PSPRS, which is indicative of overall severity of symptoms. In addition, midbrain surface is highly correlated with the PSPRS ocular motor domain, gait domain and bulbar domain sub-scores. There were no correlations between midbrain atrophy and limb domain and cognitive domain subscores. Previous studies have highlighted the correlation between midbrain atrophy and ocular-motor dysfunction [39, 40]. These findings imply that midbrain atrophy is preferentially related to symptoms characteristic of PSP-RS (i.e., ocular motor dysfunction, gait difficulties) and does not correlate with symptoms more commonly present in PSP variants (limb and cognitive subscores). As discussed previously, midbrain atrophy performed significantly better in PSP-RS vs. PSP variants, which is in agreement with these findings.

This study has certain limitations. An initial limitation is the lack of neuropathological confirmation of clinical diagnoses, which is expected in studies of exceedingly rare disorders. This is the major limitation of all relevant studies on MRI markers in Parkinsonian disorders, with only few studies in the field including small cohorts of neuropathologically confirmed patients. These studies have provided conflicting results regarding the diagnostic accuracy of MRI markers in neuropathologically confirmed cases [35, 41–43]. This limitation is particularly important for patients with a CBD diagnosis. Although clinical diagnostic criteria for CBD have been established, these criteria lack neuropathological specificity [44]. Thus, it is likely that a subgroup of patients fulfilling criteria for CBD in our study may in fact harbor a different pathology, most commonly PSP or Alzheimer’s disease [45]. This is an inherent limitation of the established diagnostic criteria and should be taken into account in the interpretation of our study findings. This limitation extends to patients with a clinical diagnosis of PSP [46], particularly with a possible or suggestive level of diagnostic accuracy [47] and could result in an inflation of the diagnostic accuracy of MRI markers, as evidenced by studies reporting lower diagnostic accuracy [48]. In addition, data regarding the genetic status of our cohort is lacking. None of the patients included had a positive family history of an atypical parkinsonian disorder. However, it is possible that patients with a genetic form of atypical Parkinsonism may have been included in our cohort. PSP-P patients were underrepresented in this cohort. Our study only included patients who were hospitalized in our ward and did not include data from an outpatient clinic. Since PSP-P patients often manifest with asymmetrical tremor, responsive to dopaminergic treatment, they are commonly misdiagnosed as PD patients and are thus rarely hospitalized. In addition, the limitations of volumetric measurements reported herein cannot be generalized and refer to the Freesurfer package which was utilized throughout this study. Lastly, this study was retrospective and thus did not include longitudinal data which would provide more robust information on the temporal evolution of atrophy in PSP patients. Despite the retrospective inclusion of patients, data collection is being performed in a systematized, prospective manner in our ward, in an effort to have robust clinical data.

Notwithstanding these limitations, our study provides data based on one of the largest single-center cohorts to date, with an excellent representation of all major Parkinsonian disorders (CBD, MSA, PD). Importantly, all subjects underwent an identical, standardized MRI acquisition protocol and an automated MRI pre-processing protocol (for volumetry), which is pivotal for controlling for possible confounding factors present when utilizing different MRI acquisition protocols. To our knowledge, this study represents the first effort to systematically analyze the most commonly used MRI markers of different modality in a standardized manner. Along the same lines, it systematically examines the effects of clinical phenotype, level of diagnostic certainty and disease duration on the diagnostic accuracy of these MRI markers in the diagnosis of PSP, in an effort to better comprehend the limitations and applicability of these markers in clinical practice.

In conclusion, this single-center study supports the superiority of surface-based markers, and of midbrain surface in particular, among MRI markers in the diagnosis of PSP, even at early disease stages. However, the utility of MRI markers decreases significantly in patients with a possible/suggestive clinical diagnosis of PSP and in patients with PSP variants, which is a major limitation in the application of these markers in clinical practice. These limitations emphasize the primary importance of clinical diagnosis, which can be further supported by MRI markers in certain clinical settings. However, the findings of this study need further validation, taking into consideration the retrospective, single-center design of the study and the lack of neuropathological confirmation of diagnoses. A large-scale, longitudinal, multi-center study including neuropathological confirmation of the clinical diagnoses would provide more robust insights into the complex correlations of imaging and clinical phenomena in PSP patients throughout the natural course of this rare disorder.

## Supplementary Information

Below is the link to the electronic supplementary material.

Supplementary file1 (DOCX 901 KB)

## Data Availability

The data that support the findings of this study are available from the corresponding author upon reasonable request.
