# Supplementary material for: In search of the optimal MRI marker for progressive supranuclear palsy: a large, single-center, retrospective study on the effect of phenotype, diagnostic certainty and disease duration
Source: J Neurol. 2025 Jul 21;272(8):523. doi: 10.1007/s00415-025-13262-2 (PMC12279571; doi:10.1007/s00415-025-13262-2)
Supplement: Supplementary file 1 — Supplementary file1 (DOCX 901 KB) [file 415_2025_13262_MOESM1_ESM.docx]

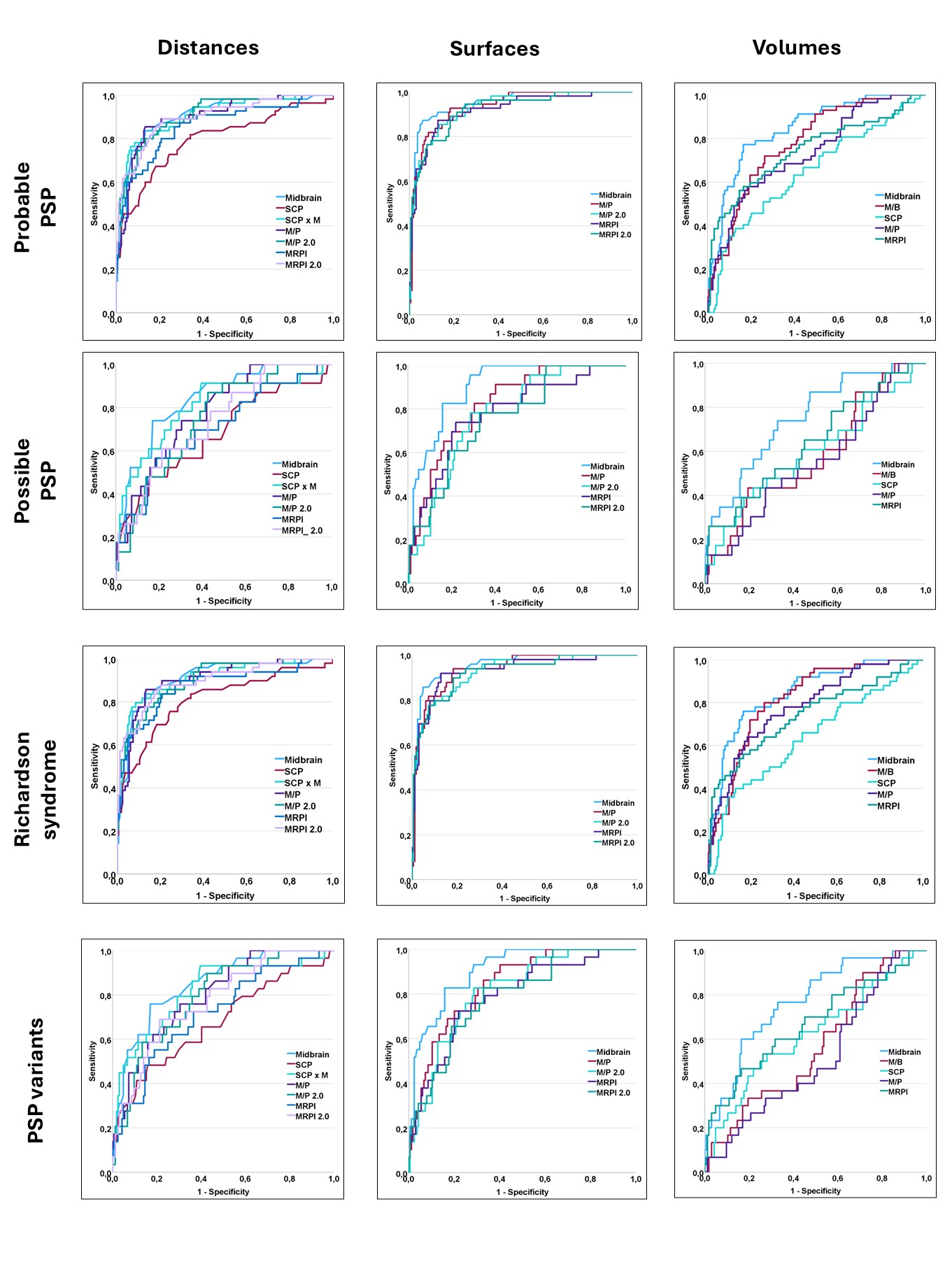


**Supplementary Figure 1.** ROC curves of MRI markers based on modality and diagnostic certainty


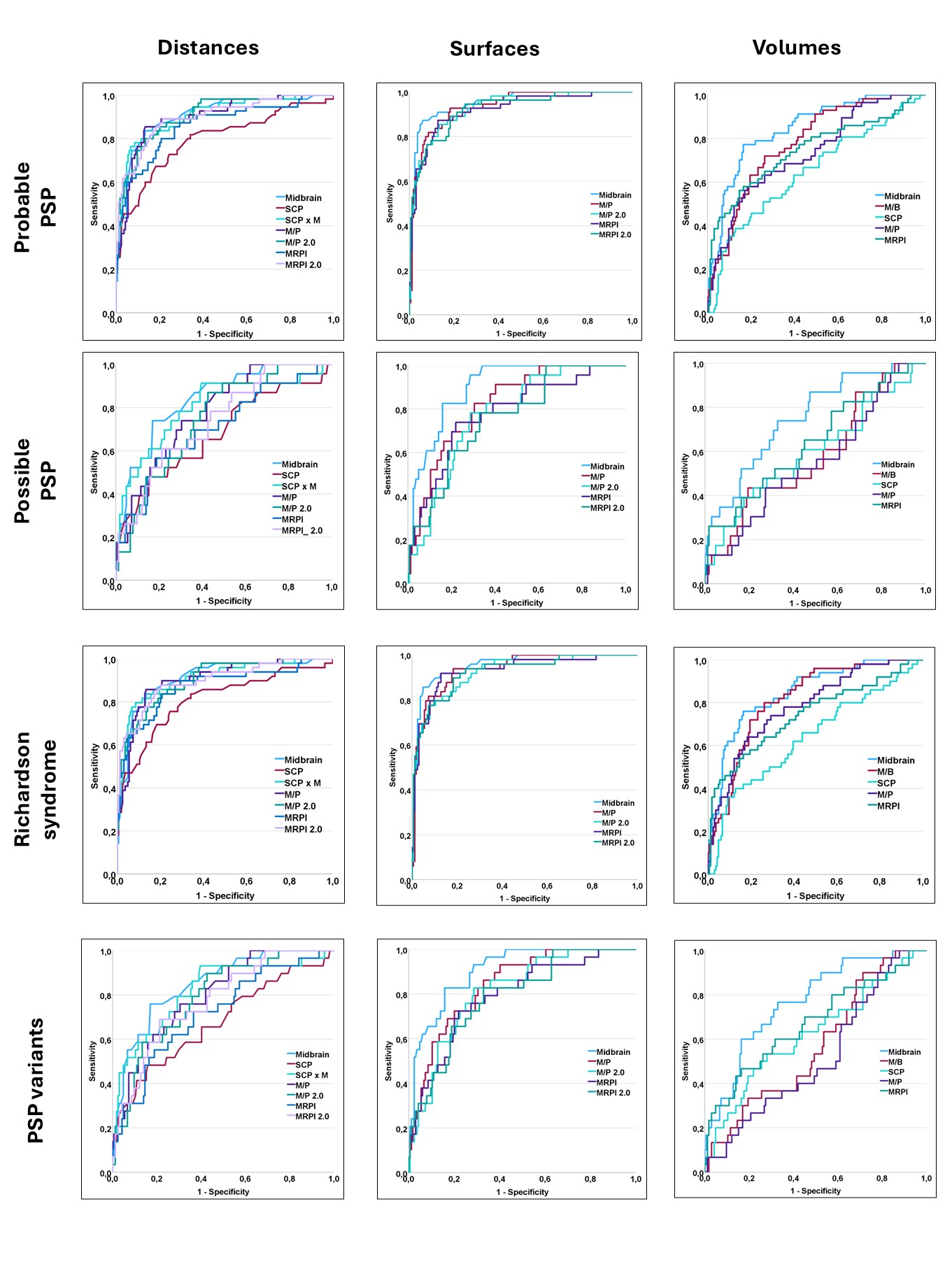


**Supplementary Figure 2.** ROC curves of MRI markers based on clinical presentation (PSP-RS vs. PSP variants)


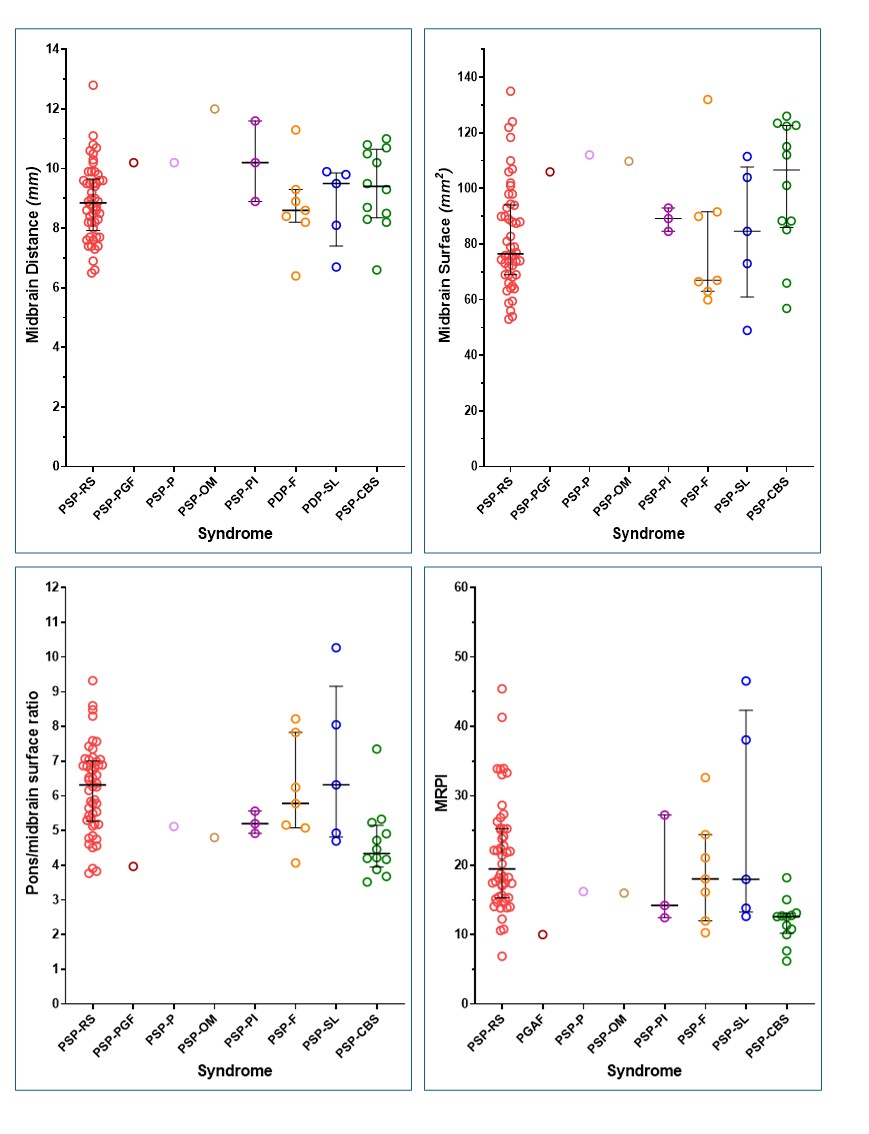


**Supplementary Figure 3.** Scatterplots with median, interquartile range per PSP phenotype of: a) midbrain anterior-posterior distance; b) midbrain surface; c) pons/midbrain surface ratio; d) MRPI_s._


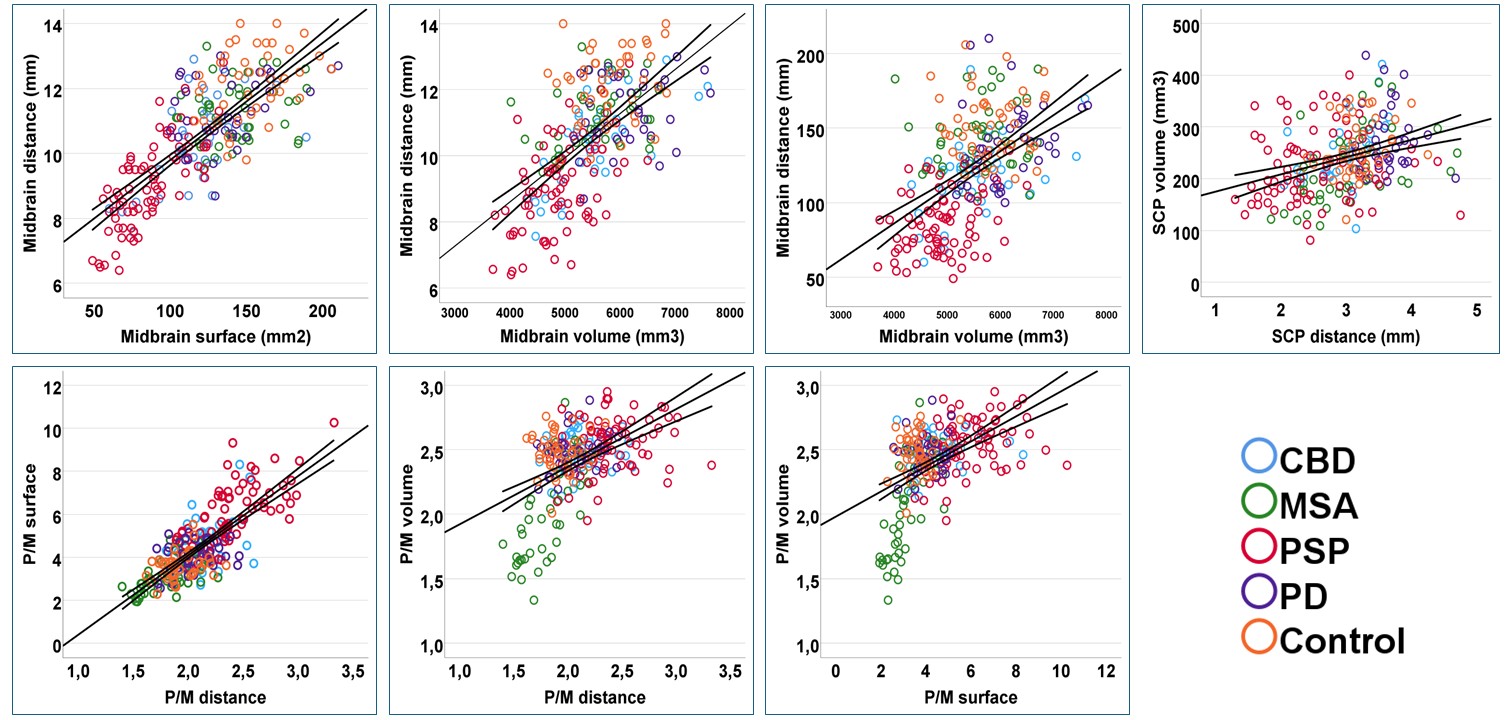


**Supplementary Figure 4.** Scatterplots of correlations between MRI markers: a) M_d_ vs. M_s_; b) M_s_ vs. M_v_; c) M_s_ vs. M_v_; d) SCP_d_ vs. SCP_v_; e) P/M_d_ vs. P/M_s_; f) P/M_d_ vs. P/M_v_; g) P/M_s_ vs. P/M_v_.


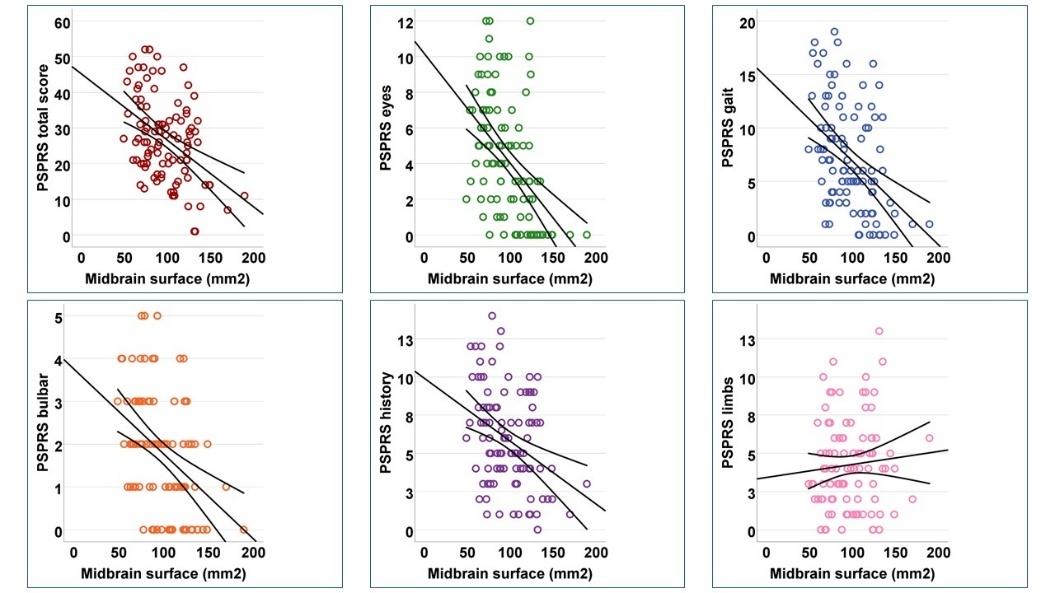


**Supplementary Figure 5.** Scatterplots of correlations between midbrain surface and PSPRS total score (a), eye subscore (b), gait subscore (c), bulbar subscore (d), history subscore (e), and limb subscore (f).
